# Recurrent Rhesus alloimmunization with early-onset severe fetal anaemia in a low-resource setting: a case report

**DOI:** 10.11604/pamj.2026.53.167.51257

**Published:** 2026-04-20

**Authors:** Willis Okumu Ochieng, Rosa Ndiema Chemwey, Elijah Kagondu Thagana

**Affiliations:** 1 Department of Obstetrics and Gynaecology, University of Nairobi, Nairobi, Kenya,; 2 Department of Obstetrics and Gynaecology, Kenyatta National Hospital, Nairobi, Kenya

**Keywords:** Rhesus alloimmunization, fetal anaemia, intrauterine transfusion, middle cerebral artery-peak systolic velocity, case report

## Abstract

Fetal anaemia due to Rhesus alloimmunization remains a significant cause of perinatal mortality and morbidity in low- and middle-income countries (LMICs), where the unaffordability of anti-D immunoglobulin compromises the care of Rhesus-negative women. We report a case of a 37-year-old multiparous RhD-negative woman who presented at 18+2 weeks of gestation with a history of recurrent fetal losses attributed to recurrent Rhesus alloimmunization. Serial fetal cerebral blood flow assessments demonstrated progressively worsening anaemia, prompting two intrauterine transfusions. Despite intervention, intrauterine fetal demise still occurred. This case highlights the aggressive course of recurrent Rhesus alloimmunization and the challenges of management in a low-resource setting.

## Introduction

Rhesus alloimmunization is an important cause of fetal anaemia and perinatal morbidity and mortality, particularly in LMICs, where access to anti-D immunoglobulin remains limited [1-3]. Following a sensitizing event in a Rh-negative woman, maternal hemolytic IgG antibodies cross the placenta and cause hemolytic disease of the newborn and fetus (HDFN), which may result in fetal hydrops, high-output cardiac failure, and perinatal death [1-5]. Fetal anaemia can also result from congenital infections, including cytomegalovirus, toxoplasmosis, and syphilis [4]. The severity of HDFN increases in subsequent pregnancies, particularly in women with antibodies detected late during the initial sensitized pregnancy [6]. The magnitude of fetal hemolysis is determined by the titers of maternal hemolytic antibodies and the history of previously affected pregnancies. Non-invasive assessment of the fetal middle cerebral artery-peak systolic velocity (MCA-PSV), expressed as multiples of the median (MoM), is the standard tool for predicting and monitoring fetal anaemia. The optimal MCA-PSV threshold value for moderate-to-severe fetal anaemia requiring intervention is 1.50 MoM. Intrauterine transfusion remains the mainstay of treatment for fetal anaemia [3,4,7-10]. We report a case of recurrent Rhesus alloimmunization with early-onset severe fetal anaemia managed in a low-resource setting.

## Patient and observation

**Patient information:** a 37-year-old para 5+1 gravida 7 RhD-negative woman presented at 18+2 weeks with a history of recurrent fetal losses. One pregnancy had been complicated by hypertensive disease, while the other five were associated with hydrops fetalis attributed to maternal Rhesus alloimmunization. There was no documented history of anti-D prophylaxis in her previous pregnancies. Her current partner, who is blood group O RhD-positive, is the same as in all previous pregnancies. She reported never having been transfused before.

**Clinical findings:** on examination, she was in a fair general condition with stable vital signs. Systemic examination was unremarkable, and fundal height corresponded to the gestational age.

**Figure 1:**
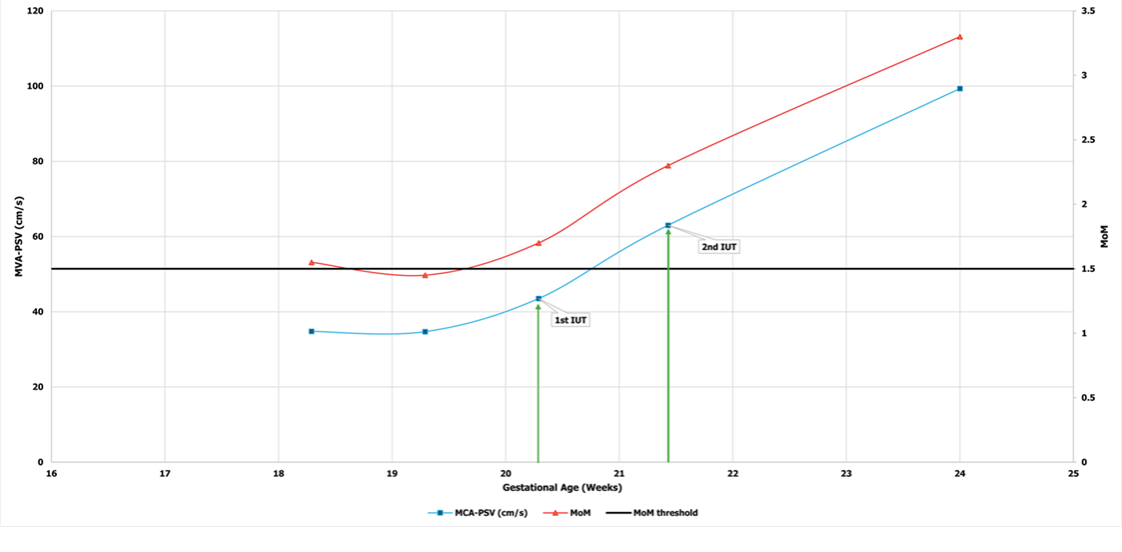
fetal middle cerebral artery peak systolic velocity (MCA-PSV) and corresponding multiples of the median (MoM) trend across gestation; the continuous black line represents the threshold of 1.5 MoM for moderate-to-severe fetal anaemia; green arrows indicate the gestational ages at which the intrauterine transfusions (IUT) were performed

**Timeline of current episode:** from 18+2 weeks, MCA-PSV measurements were obtained and averaged. The corresponding MoMs were calculated using Mari's formula [9]. Middle cerebral artery-peak systolic velocity was 34.78 cm/s (1.55 MoM) and 34.69 cm/s (1.45 MoM) at 18+2 and 19+2 weeks, respectively, and rose to 43.49 cm/s (1.7 MoM) by 20+2 weeks. At 20+4 weeks, intrauterine intraperitoneal transfusion (IUIPT) was performed. Follow-up MCA-PSV increased to 62.98 cm/s (2.3 MoM) at 21+3 weeks, and an intrauterine intravascular transfusion (IUIVT) was performed at 21+4 weeks. At 24+0 weeks, MCA-PSV further rose to 99.33 cm/s (3.3 MoM). A follow-up ultrasound scan done ten days later showed intrauterine fetal demise at 25+4 weeks. These trends are highlighted in Table 1 and Figure 1.

**Table 1:** serial fetal middle cerebral artery peak systolic velocity measurements and sonographic findings in a 37-year-old RhD-negative woman with severe fetal anaemia due to recurrent rhesus alloimmunization

Gestational age (weeks)	Middle cerebral artery-peak systolic velocity (MCA-PSV) (cm/s)	Multiples of the median (MoM)	Other sonographic findings
18+2	34.78	1.55	Normal growth and gross fetal morphology Normal fetal echocardiography and neurosonogram Normal facial profile; bilateral echogenic kidneys Bladder visualized Absent markers for aneuploidy
19+2	34.69	1.45	Early anatomy: normal neuroanatomy, spine, upper and lower limbs Fetal echocardiography and uterine artery Doppler studies normal
20+2	43.49	1.70	Appropriate growth Normal anatomy for gestation
20+4	-	-	First intrauterine transfusion (13 ml PRBCs)
21+3	62.98	2.30	Appropriate growth Normal anatomy for gestation
21+4	-	-	Second intrauterine transfusion (30 ml PRBCs)
24+0	99.33	3.30	Appropriate growth Normal anatomy for gestation
25+4	-	-	Intrauterine fetal demise

**Figure 2:**
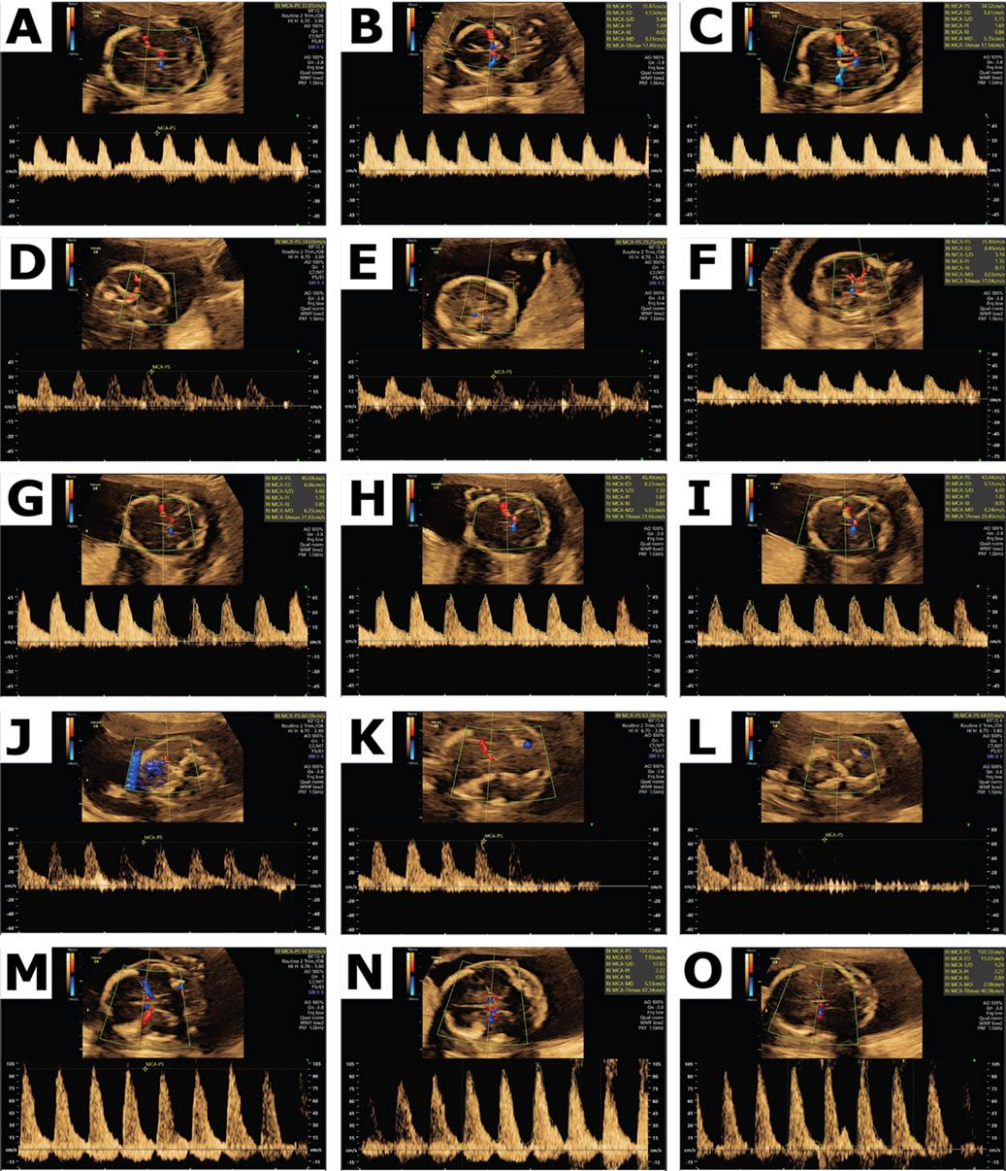
representative Doppler images demonstrating fetal middle cerebral artery peak systolic velocity (MCA-PSV) measurements across gestation: A-C) 18+2 weeks, MCA-PSV 34.78 cm/s (1.55 MoM); D-F) 19+2 weeks, MCA-PSV 34.69 cm/s (1.45 MoM); G-I) 20+2 weeks, MCA-PSV 43.49 cm/s (1.7 MoM); J-L) 21+3 weeks, MCA-PSV 62.98 cm/s (2.3 MoM); M-O) 24+0 weeks, MCA-PSV 99.33 cm/s (3.3 MoM)

**Diagnostic assessment:** her blood group was B RhD-negative, and antibody screening showed an indirect Coombs titer of 1:32. Although tests to rule out potential etiologies of fetal anemia such as alloimmunization by atypical antigens, congenital infections, and inherited conditions such as hemoglobinopathies were not performed due to financial constraints, there were no clinical or sonographic features suggestive of congenital infection, including absence of fetal structural anomalies or organomegaly. A singleton pregnancy was confirmed, excluding twin-related causes. In the absence of prior transfusion, alloimmunization to minor red cell antigens was considered unlikely. Serial MCA-PSV measurements demonstrated progressively rising MoMs, consistent with worsening fetal anaemia [4,8,10]. Representative Doppler findings are shown in Figure 2.

**Diagnosis:** the findings were consistent with severe fetal anaemia secondary to recurrent maternal Rhesus alloimmunization, based on the positive indirect Coombs test titer (1:32) and progressively rising MCA-PSV values exceeding 1.5 MoM [4,8,10]. The prognosis was poor given the early gestational age at presentation and rapid progression of fetal anaemia [10].

**Figure 3:**
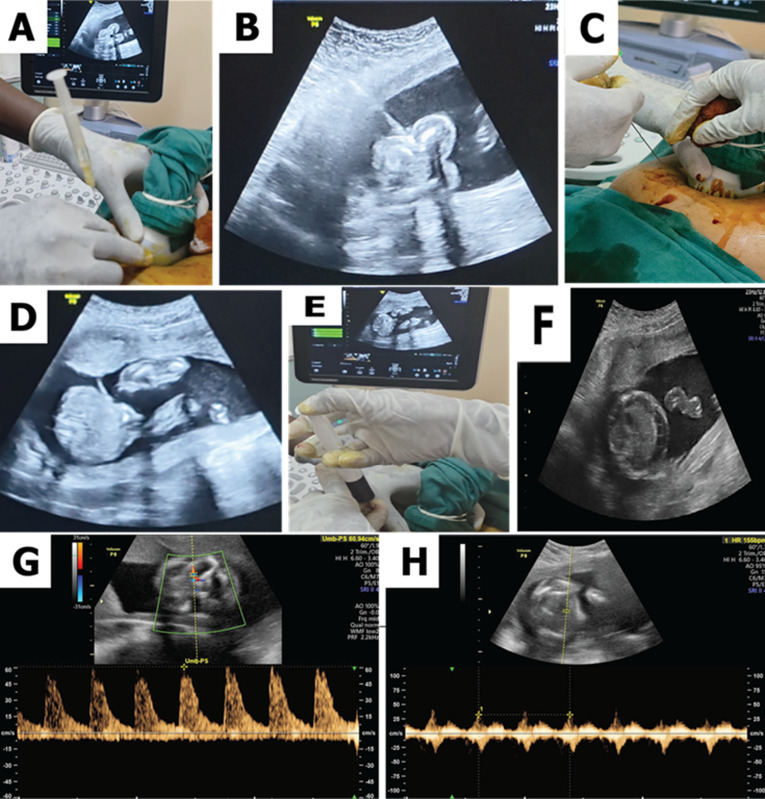
ultrasound-guided intrauterine transfusion at 21+4 weeks' gestation; A,B) fetal sedation with intramuscular atracurium; C-E) intrauterine intravascular transfusion under ultrasound guidance; F) post-transfusion wheal around the fetal liver; G-H) confirmation of fetal viability post-transfusion

**Therapeutic interventions:** MoMs remained persistently ≥ 1.5, indicating worsening fetal anaemia [4,8,10]. Intrauterine transfusion (IUT) was performed at 20+4 and 21+4 weeks using leukoreduced, O-negative packed red blood cells (PRBCs) under ultrasound guidance [8,10]. The fetus was sedated with intramuscular atracurium before transfusion [3]. Fetal viability was then confirmed post-transfusion (Figure 3).

**Follow-up and outcome of interventions:** despite two IUTs, the fetus showed no improvement clinically, as MCA-PSV/MoMs continued to rise, indicating progressively worsening anaemia [4,8,10]. A follow-up obstetric ultrasound showed intrauterine fetal demise at 25+4 weeks.

**Patient perspective:** the patient understood that the pregnancy was affected by rhesus alloimmunization with severe fetal anaemia, as evidenced by the increasing MoMs. She appreciated the care that she received throughout the pregnancy. She wished that anti-D prophylaxis in her first affected pregnancy might have altered the outcome of her subsequent pregnancies. For her subsequent pregnancies, she stated that she would seek fetal-maternal counseling before conception.

**Informed consent:** written informed consent was obtained from the patient. All patient information was de-identified to protect confidentiality. Ethical approval for this case report was granted by the Kenyatta National Hospital-University of Nairobi Ethics and Research Committee (KNH-UoN ERC).

## Discussion

Recurrent Rhesus alloimmunization with early-onset progressive fetal anaemia is a rare and challenging obstetric complication, particularly in LMICs, where resource limitations may hinder prevention and management [1,2,6]. Following a sensitizing event, maternal hemolytic IgG antibodies are produced and traverse the placenta, causing fetal anaemia [1-5]. Severe HDFN in subsequent pregnancies demonstrates a stronger antibody response in those who have never received anti-D prophylaxis [6]. This aligns with our patient´s history of recurrent fetal losses with no documented anti-D prophylaxis in the preceding pregnancies. Fetal anaemia may cause fetal hydrops, high-output cardiac failure, and perinatal death [1-5]. In this case, maternal anti-D titers of 1:32, combined with the history of previously affected pregnancies, predisposed the fetus to significant anaemia. Serial fetal MCA-PSV measurements confirmed the progression of anaemia, with persistently elevated MoM of ≥ 1.5 consistent with moderate-to-severe fetal anaemia [3,4,7,8,10].

Studies have shown that fetal blood viscosity, which determines the MCA-PSV/MoM, and thus its sensitivity in predicting the optimal timing of subsequent transfusions, is altered after IUT [8]. This is consistent with our case where anaemia progressed despite intervention. Despite studies showing that ultrasound-guided IUT improves the treatment of early severe fetal anaemia and survival rates of affected fetuses, this wasn´t the result in our case. Similar outcomes have been encountered in high-resource settings where early fetal transfusions before 18 - 20 weeks´ gestation have been found to have low success rates. Although intravascular transfusion is superior to intraperitoneal transfusion, the latter was performed initially in this case due to the small size of the fetal structures and limited visualization [1,3,4,8,10]. Thus, the adverse outcome in our case reflects the severity of HDFN and systemic limitations, rather than delayed clinical decision-making. Survival rates following intrauterine transfusion are close to 90%; however, outcomes are poorer when intervention is required early in gestation, as demonstrated in this case [4,10].

Several alternative causes of fetal anemia were considered. Minor maternal antibodies against other red cell antigens (Cc, D, Ee, Kell, Duffy, Kidd, Jka, Jkb, and M) can lead to HDFN, but these were unlikely due to the absence of prior transfusions, and the strong correlation of maternal anti-D titers with disease severity [2,4,6,10]. Fetal parvovirus B19, which suppresses erythropoiesis, as well as cytomegalovirus, syphilis, and other TORCHES (Toxoplasmosis, Other infections, Rubella, Cytomegalovirus, Herpes) infections were also considered but excluded based on the clinical course and normal structural ultrasound findings [10]. Hemoglobinopathies were unlikely in the absence of a suggestive maternal history or sonographic findings. Conditions such as twin-to-twin transfusion syndrome or twin anaemia-polycythemia sequence were ruled out by confirming a singleton pregnancy [4,10]. The combination of recurrent pregnancy losses, high anti-D titers, and progressive MCA-PSV changes strongly support recurrent Rhesus alloimmunization as the primary etiology [6,8-10]. This case also highlights the challenges unique to LMICs, which compromise the obstetric care of a Rhesus-negative woman. Unaffordability of anti-D immunoglobulin, poor documentation of potentially sensitizing events, delayed referrals to specialized care, and lack of advanced fetal therapies contribute to poor outcomes [1-3].

## Conclusion

This case describes recurrent Rhesus alloimmunization presenting with early-onset severe fetal anaemia and poor fetal outcome despite intrauterine transfusion in a low-resource setting. It highlights the aggressive nature of the disease when sensitization occurs without prior anti-D prophylaxis and demonstrates the limitations of available interventions when anaemia develops early in gestation. Early identification of at-risk pregnancies, timely administration of anti-D immunoglobulin, and prompt referral for specialized maternal-fetal care are essential to improve outcomes.
